# Role of exosomal noncoding RNA in esophageal carcinoma

**DOI:** 10.3389/fonc.2023.1126890

**Published:** 2023-05-09

**Authors:** Mao-Yan Si, Ding-Yu Rao, Yao Xia, Cheng-Peng Sang, Kai-Yun Mao, Xiang-Jin Liu, Zu-Xiong Zhang, Zhi-Xian Tang

**Affiliations:** ^1^ First Clinical Medical College, Gannan Medical University, Ganzhou, China; ^2^ Department of Cardiothoracic Surgery, The First Affiliated Hospital of Gannan Medical University, Ganzhou, China; ^3^ Department of Cardiothoracic Surgery, The Affiliated Huaian Hospital of Xuzhou Medical University, Huai’an, Jiangsu, China

**Keywords:** esophageal cancer, exosomes, IncRNA, miRNA, prospects

## Abstract

Esophageal cancer is a common malignant tumor with a high degree of malignancy. Understanding its pathogenesis and identifying early diagnostic biomarkers can significantly improve the prognosis of esophageal cancer patients. Exosomes are small double-membrane vesicles found in various body fluids containing various components (DNA, RNA, and proteins) that mediate intercellular signal communication. Non-coding RNAs are a class of gene transcription products that encode polypeptide functions and are widely detected in exosomes. There is growing evidence that exosomal non-coding RNAs are involved in cancer growth, metastasis and angiogenesis, and can also be used as diagnostic and prognostic markers. This article reviews the recent progress in exosomal non-coding RNAs in esophageal cancer, including research progress, diagnostic value, proliferation, migration, invasion, and drug resistance, provide new ideas for the precise treatment of esophageal cancer.

## 1 Introduction

Esophageal cancer (EC) is one of the malignant tumors with high incidence and a serious threat to human health and life worldwide. Although significant progress has been made in the diagnosis, treatment, and pathogenesis of esophageal cancer for more than half a century, the five-year survival rate is still less than 20% (1). Moreover, its incidence and mortality continue to increase each year. The EC pathogenesis has not been clarified. The morbidity and mortality may be related to many factors. In recent years, many epidemiological studies have shown that esophageal cancer occurrence results from long-term interaction between genetic and environmental factors (2, 3). Because most esophageal cancer patients are in the middle and advanced stages of the disease when they are treated, the treatment methods are very limited (4). The primary treatment for esophageal cancer is surgery (5, 6). Even after rigorous surgical treatment, EC patients’ leading causes of death are tumor recurrence and distant metastases. New noninvasive biomarkers and therapeutic targets must be identified to improve esophageal cancer patients’ survival rate and quality of life.

Exosomes are a type of microvesicle with a size of 40~100 nm that are widely distributed in bodily fluids such as blood, saliva, and urine and are secreted into the circulatory system by cells. The vesicle carries various biological information molecules, such as mRNA, miRNA, and DNA fragments (7) which are often related to carcinogenesis, invasion, and metastasis (8). In recent years, detecting specific information molecules in exosomes for diagnosing malignant cancers have become a research priority.

**Figure 1:**
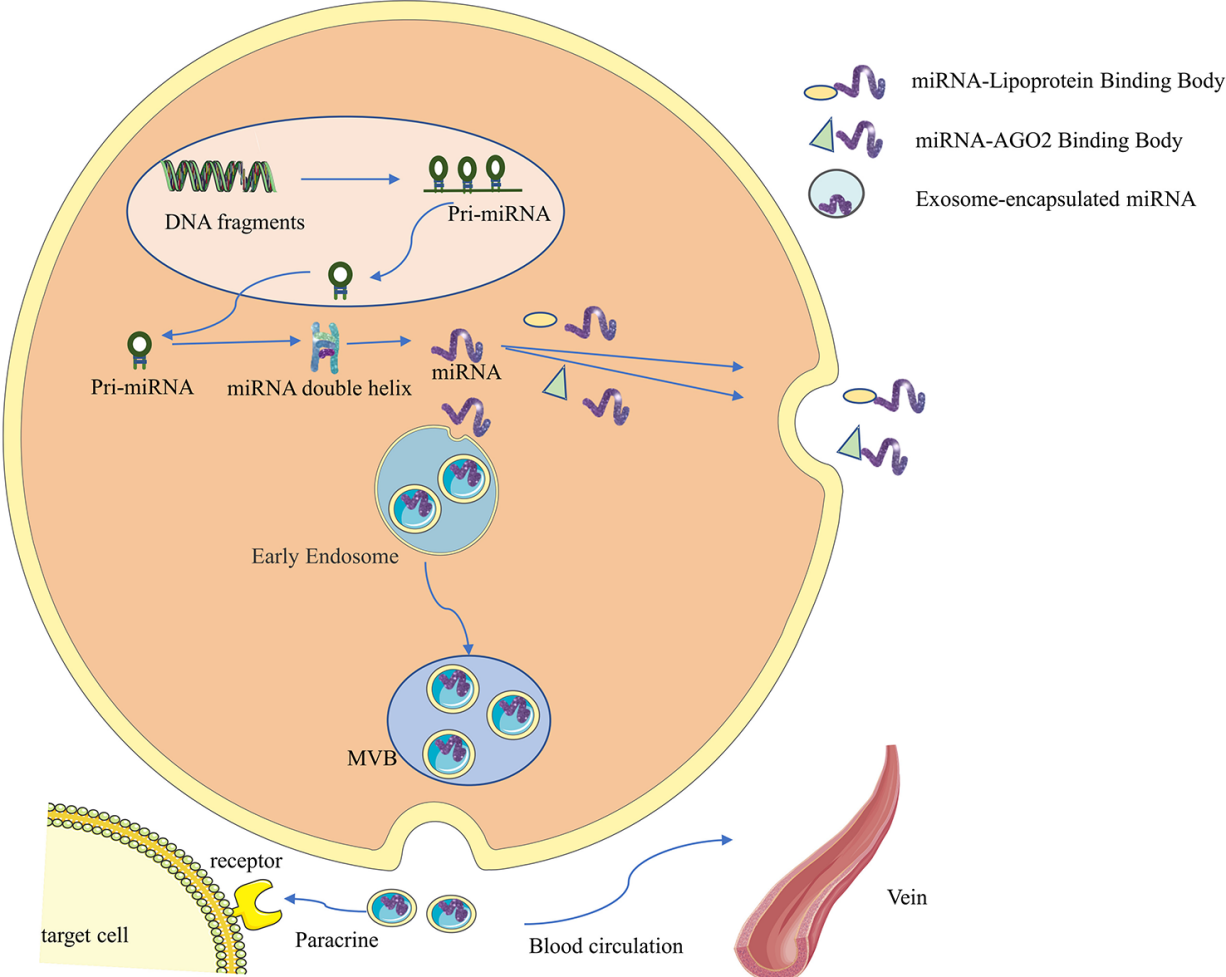
Synthesis, secretion and functional characteristics of exosomal miRNA. First, miRNA genes are transcribed into primary miRNAs (pri-miRNAs) in the nucleus, which range in length from hundreds to thousands of bases and contain one to several hairpin stem-loop structures with a 5’cap and a 3’polyA tail. The pri-miRNA is then further processed by the nuclease Drosha and its cofactor Pasha to form a precursor miRNA (pre-miRNA), consisting of 70 nucleotides, which is then transported into the cytoplasm *via* the GTP-dependent Exprotin-5 complex. Finally, the pre-miRNA is further cleaved by Dicer enzyme to form double-stranded miRNA:miRNA, then one miRNA chain is degraded, and the other mature miRNA chain binds to the 3’UTR of the target mRNA, so that the target mRNA is degraded or translation is inhibited, so as to achieve the purpose of regulating protein expression. miRNAs do not circulate in the body in a free state. They generally bind to AGO2 or lipoproteins, or are encapsulated by vesicles such as exosomes before entering the circulation.

Non-coding RNAs (ncRNAs) used to be considered as a class of gene transcription products that do not possess protein-coding functions, but more and more studies have shown that some ncRNAs can encode to produce functional polypeptides (9), and such ncRNAs and polypeptides can be confirmed to often have high conservation and homology by sequencing and mass spectrometry. Among them, lncRNAs and circRNAs can participate in regulating transcription and translation processes directly by performing functions such as protein scaffolding, regulatory splicing, and loop-roll translation, or indirectly regulating signaling pathways by influencing other RNAs as miRNA sponges, thus participating in regulating tumorigenesis and development (10–12). ncRNAs are widely found in exosomes (13–15). In addition, miRNAs do not circulate in the body in a free state; they generally bind to AGO2 or lipoproteins or are encapsulated by vesicles such as exosomes before entering the circulation (as shown in **Figure 1**) (5). Compared to miRNAs that bind to proteins and circulate *in vivo*, exosomal miRNAs may have better structural stability, target specificity and functional direction.

Many studies have found tumor-derived exosomes in the circulating blood of esophageal cancer patients (16–18). These exosomes include numerous tumor-related specific molecules, such as mRNA, protein, lipid, and non-coding RNA., making them a powerful signal transmission function (19). Due to the necessity for numerous signal exchanges between the tumor and surrounding supporting cells, the exosome secretion increases significantly, participating in the regulation of tumor microenvironment and metastasis, and might play a key role in it. The analysis and detection of tumor exosomes can assist in early diagnosis, efficacy evaluation and prognosis analysis of tumors (20). For example, in esophageal squamous cell carcinoma, high miRNA-21 expression in exosomes can reflect 100% of the tumor level. Moreover, high miRNA-21 expression in exosomes often indicates extensive invasion and recurrence (21).

## 2 Exosomes research progress

Studies of exosomes date back to 1946 when Chargaff and West reported that plasma clotting time increased after ultracentrifugation (22). The researchers attributed this phenomenon to subcellular procoagulant factors, which are small lipid-rich vesicles with 20 to 50 nm in diameter; in 1967, Wolf called it “platelet dust” (23). Endocytic vesicles were first identified in mature mammalian reticular cells (immature erythrocytes) in 1983 by Stahl’s (24) and Johnstone’s teams (25). In 1987, Johnstone et al. (26) defined vesicles formed in multivesicular bodies by endocytosis and released by the fusion of multivesicular bodies with plasma membrane as “exosomes”. In the following decade, exosomes were not taken seriously by researchers. In 1996, Raposo et al. (27) reported that exosomes secreted from B lymphocytes, which carry MHC class II molecules, costimulatory factors, and adhesion factors, could represent antigens. Studies have shown that these B cell-derived exosomes can directly stimulate the antitumor response of CD4+ cells. In 2007, Valadi et al. (28) found that RNA can be exchanged between different cells through exosomes and confirmed that tumor exosomes could promote or inhibit the growth and metastasis of tumor cells. The 2013 Nobel Prize in Physiology or Medicine was awarded by United States scientist James E. Rothman and Randy W.Schekman, Thomas C. Three scientists from Südho were awarded for their discovery of the regulatory mechanism of vesicular trafficking, the main transport system in cells, which pushed exosomes to a new climax. However, during the past decade, significant advancements have been achieved in this field of study, particularly the discovery of exosomal miRNA activity, which is crucial in cancer research. Therefore, exosomes can be used as diagnostic markers and prognostic indicators for tumors (29). Due to its characteristics can also be used as a carrier for drugs and functional molecules, providing a novel clinical therapeutic mode (30).

## 3 Diagnostic value of exosomal ncRNA in EC

The “Asian esophageal cancer belt” extends from northern Iran through the Central Asian republics to Mongolia and north-central China. It is a special high-risk area for Esophageal squamous cell carcinoma (ESCC), with China alone accounting for more than half of the global cases (31). Because the early symptoms of EC are not obvious, patients are often in the middle and late stages when they are diagnosed, severely impacting their families and daily lives (32). Therefore, early detection and prompt treatment of esophageal cancer are of clinical importance (33). There is increasing evidence that early diagnosis and accurate prediction of treatment effects can significantly improve the ESCC patient’s prognosis (34). However, the specificity and sensitivity of its diagnostic and prognostic biomarkers remain unsatisfactory. Numerous studies have shown that cancer cell-derived exosomes contain specific nucleic acids and proteins that reflect the cancer cells’ origin (35). Therefore, exosomes are novel and potential biomarkers in many cancer types (36). Compared to other cancer biomarkers (such as circulating tumor cells (CTC) and circulating tumor DNA (ctDNA), exosomes have the advantages of sufficient quantity, strong stability, and strong accessibility (37, 38). Almost all cancer cell types can secrete numerous exosomes, and exosomes exist in almost all body fluids, such as blood, saliva, urine, tissue fluid and cerebrospinal fluid, which broadens the selection range of liquid biopsy sample sources (37, 39).

### 3.1 Exosomal miRNA in EC

Lin et al. (40) showed the presence of miRNA in EC patients’ saliva and animal saliva exosomes. Furthermore, the chimeric RNA level in saliva exosomes can be used for the first time as a noninvasive biomarker for detecting early and late EC for postoperative monitoring, therapeutic response, and tumor recurrence (41).

The study of Li et al. (37), by comparing small RNAs in salivary exosomes from ESCC patients with RNA from healthy controls, a cancer-rich dual sesncRNA profile (i.e., tRNA-GlyGCC-5 and sRESE) was identified in salivary exosomes, which represents a non-invasive, convenient, and reliable biomarker for human ESCC diagnosis, prognosis, and especially prediction of preoperative patients who may benefit from adjuvant therapy.

Furthermore, samples from 51 patients with ESCC and 41 patients with benign illnesses were collected (i.e., the control group). Exosomal miR-21 levels were significantly increased in ESCC groups compared to controls (42). Exosome miR-21 may be a useful biomarker for detecting ESCC progression or efficacy of ESCC.

### 3.2 Exosomal IncRNA in EC

Yan et al. (43) found that serum exosomal lncRNA can be used as a biomarker for diagnosing and prognosis of EC. Furthermore, the lncRNA of four UCA1, ESCCAL-1, PEG10 and POU3F3 was most significantly up-regulated in EC exosomes. Similarly, increased expression levels are also observed in patients with advanced disease stages. Using ROC analysis, some lncRNAs showed high diagnostic values, e.g. AUCs for UCA1 and POU3F3 were 0.733 and 0.717, respectively. Based on these findings, the exosomal lncRNA combination provides a more sensitive diagnosis of ESCC, particularly for early disease. In Huang et al. (44), lncRNA PCAT1 was present in ESCC cell-derived exosomes and upregulated in the serum of ESCC patients. Further studies have shown that PCAT1 is an oncogene in ESCC and promotes ESCC progression by binding to miR-326. PCAT1 can be used as a therapeutic target and a potential non-invasive biomarker for ESCC patients.

## 4 Influence of exosomal ncRNA on migration, and invasion of EC

In the field of tumor research, there have been considerable literature reports that exosomes can participate in many tumors’ progression, including promoting tumor proliferation, metastasis, and invasion (45, 46), inhibiting tumor cell apoptosis (47), regulating cell cycle (48, 49) and autophagy (50). The tumor-derived exosomal lncRNA ZFAS1 promotes proliferation. It inhibits apoptosis by up-regulating STAT3 and down-regulating miR-124, thus benefiting ESCC cells’ tumorigenesis (8). Tumor-derived exosomal miR-19b-3p can target Chromosome 10 promote EC cell invasion and inhibit apoptosis (51). Matsumoto et al. demonstrated that tumor-derived exosomes (52)could promote tumor progression and malignant transformation by altering gene expression and tumor cell phenotype (48). For example, Tumor-derived exosomal lncRNA PCAT1 (prostate cancer-associated transcript 1) promote ESCC cell proliferation *via* the sponge tumor suppressor miR-326 (44).

Cancer-associated fibroblasts (CAFs) are one of the most important components of the tumor microenvironment and play an essential role in tumor occurrence and development (53). Similarly, CAFs have been shown to contribute to tumor development and progression (52, 54). Zhao et al. found that CAF-derived exosomes could improve the ESCC cells’ growth and migration through the Hedgehog signaling pathway (55). Furthermore, CAFs use the exosomal miR-451 as a signaling molecule, providing a favorable niche for tumor cell migration and cancer progression (56).

Tumor-associated macrophages (TAM) are infiltrating macrophages in tumor tissues. The researchers found that exosomes secreted by ESCC cells can induce macrophage polarization to the M2 type through its content miR-301a-3p. Moreover, the TAMs proangiogenic switch is triggered by exosomes miR-301a-3p secreted by ESCC cells through PTEN/PI3K/AKT signaling pathway (57). These studies highlight the important role of exosomes in the growth and migration of EC.

## 5 Exosomal ncRNA promote drug resistance in ESCC

Chemoradiotherapy is one of the most common treatments for advanced esophageal cancer, and chemotherapy resistance signifies chemotherapy failure. Therefore, the mechanism of human chemoradiotherapy resistance and how to reverse chemoradiotherapy resistance are pressing issues that must be resolved in tumor treatment. In tumors, most exosomes are tumor development promoters (58–60). Exosomes can serve as tumor signaling vectors. One of the adverse clinical impacts of exosomes as a source of tumors is their capacity to transfer resistance horizontally (61–63). Drug-resistant tumor cells can transfer drug resistance to sensitive cells through exosomes (18, 64), thus generating new anti-tumor cell reservoirs. Exosomes from drug-resistant tumor cells may confer resistance phenotype to sensitive cells through intercellular signaling.

Kang et al. found that exosomes from gefitinib-resistant cells containing the long non-coding RNA lncRNA PART1 promoted gefitinib resistance in ESCC *via* the miR-129/blc-2 axis (65). Exosomes containing miR-21 from cisplatin-resistant cells promote the development of cisplatin resistance in ESCC by targeting programmed cell death 4 (PDCD4) (66).Additionally, exosomal miR-193 delivery to ESCC cells increases cisplatin resistance by activating the janus kinase (JAK)-STAT signaling pathway (67). Furthermore, ESCC-derived exosomal lncRNA POU3F3 transforms fibroblasts (NF) into CAFs, and these CAFs can secreted IL-6 then enhances cisplatin resistance in ESCC cells (68). A recent study found that the hypoxic tumor cell-derived exosomal miR-340-5p confers radioresistance in ESCC by targeting KLF10/UVRAG (69). The miR-340-5p level in plasma exosomes is closely related to radiotherapy response and prognosis. MiR-340-5p may be a therapeutic target for overcoming radioresistance in ESCC. Luo et al. demonstrated that tumor-derived exosomal miR-339-5p enhanced the radiosensitivity of ESCC cells by targeting Cdc25A (70). These studies suggest that exosomes are vital in regulating resistance to EC therapy. It is believed that with further research, the role and mechanism of exosomes in esophageal cancer resistance will be gradually revealed and finally applied to clinical practice.

## 6 Targeted delivery of modified exosomes and their prospects

**Figure 2:**
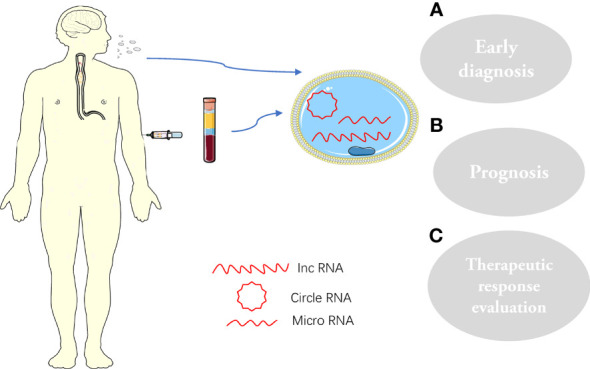
Exosomes and their cargoes, extracted from human plasma or saliva, are widely involved in the pathophysiology of ESCC: **(A)** Early diagnosis; **(B)** prognosis; **(C)** Therapeutic response evaluation.

Exosomes are widely distributed and can shuttle freely in the body, known as the “Trojan Horse”. Exosomes have a role in various physiological and metabolic processes in the body, as they facilitate the flow of information between cells (71–73) (**Figure 2**). Meanwhile, exosomes also have the characteristics of non-immunity and easy penetration of cell membranes and can be specifically recognized by receptor cells (74). Therefore, exosomes have unique natural advantages as drug delivery vehicles (75). Research on drug delivery by exosomes has become a hot spot in recent years. Some small-molecule chemical and gene drugs have been successfully loaded into exosomes, showing great potential in treating various cancers (76, 77).

Researchers at Oxford University have used exosomes as carriers to load therapeutic siRNA for treating Alzheimer’s disease. By modifying exosomes to have specific cell targeting, they not only successfully passed the blood-brain barrier but also accurately delivered therapeutic siRNA to target cells, reducing the mRNA and protein expression levels of corresponding genes in target cells, thus achieving the purpose of disease treatment (78, 79).

Exosomes are vital as drug carriers in enhancing anti-cancer response and targeted drug delivery (77, 80, 81). Exosomes can transport small molecules, such as nucleic acids, to target cells, and there are increasing studies using exosomes as vectors to deliver therapeutic nucleic acids for cancer treatment (82, 83). As a natural RNA vector, exosomes have high circulating stability and inherent homing ability, which has the advantage of simultaneous loading of multiple therapeutic nucleic acids compared with conventional antitumor delivery systems (84). Shtam et al. reported using exosomes to deliver latent therapeutic siRNAs against cancer cells to target cells. The successful delivery of siRNA to recipient cells was observed using confocal microscopy and flow cytometry. The significant reduction of oncogene protein levels and the mass death of cancer cells further proved that siRNA could be effectively delivered to target cells (85).In addition, Adriamycin-and paclitaxel-loaded exosomes have been used in cancer therapy with low immunogenicity and toxicity (86, 87). The utility of paclitaxel-loaded exosomes has improved efficacy in treating multidrug-resistant cancer cells (80). Cumulatively the above studies indicate that exosomes are an effective tool for carrying and delivering anticancer drugs.

## 7 Discussion

In this article, we attempt to summarize the exosomal ncRNAs’ role in the diagnosis, growth, metastasis, drug resistance and targeted delivery of EC. Additionally, we discussed using exosomal ncRNA as biomarkers and therapeutic tools for the diagnosis, prognosis and prediction of EC.

Although exosomal ncRNA has considerable application potential, challenges prevent its practicality. First, clinical samples require more accurate and standardized purification methods. Secondly, there are multiple bioactivators in exosomes and their main functional components still need further study. Currently, there is no standardized method for isolating and identifying exosome from biological body fluids. The methods used in the reported study lack repeatability and inconvenience, which limits their widespread use. Moreover, an ideal exosome enrichment strategy with high purity and efficiency cannot be obtained. Due to the lack of large-scale exosomes for clinical research, exosome-based engineering applications are limited to cellular or animal experiments. Finally, a systematic and in-depth study on the exocrine mechanism involved in tumor occurrence and development is lacking, Implementing exosomal ncRNA based diagnosis and treatment strategies still faces significant difficulties, but these tactics must be translated into practical application soon to assist EC patients.

## Author contributions

M-YS searched for literature and wrote the first draft of this article. D-YR edited tables and figures. Z-XZ and Z-XT strictly reviewed the manuscript and polished the grammar. All authors contributed to the article and approved the submitted version.
